# Evaluation of nerve procedures and FFMT in adult BPI - the Surabaya experience

**DOI:** 10.1186/1753-6561-9-S3-A23

**Published:** 2015-05-19

**Authors:** Heri Suroto

**Affiliations:** 1 Department of Orthopaedics, Dr Soetomo General Hospital, 6-8, Surabaya, Indonesia

## Purpose

The purpose of this study was to describe our experience with various nerve and muscle procedures in traumatic adult brachial plexus injuries.

## Materials and methods

Two hundred and nine (n=209) cases of adult brachial plexus injuries were treated in Surabaya from March 2005 to May 2014. Thirty seven cases treated in 2014 were excluded from this study.

## Results

The number of operative cases of brachial plexus injury increase each year. Fifty nine percent of cases were managed in public hospitals and 41% in private hospitals. Most of the patients were males (86%) and the rest were females (14%). Brachial plexus injury was most prevalent between 21-30 years of age (37%). Motor vehicle accident was the most frequent mode of injury (90%). The right side was the most frequently affected side (77%). The levels of injury were as follows: C5-6 postganglionic (24%), C5-7 postganglionic (19%), C8-T1 postganglionic (3%), C5-T1 (54%). In complete BPI, the combination of C5-7 postganglionic and C8-T1 preganglionic were present in 33% of cases. There were 2 types of surgical intervention: nerve procedure (67%) and muscle procedure (33%). Nerve transfer was the most frequently employed mode of nerve procedures (54%, one third of which was either Oberlin’s transfer or double fascicular nerve transfer). The most frequently performed muscle procedure was the free functioning muscle transfer (78%).

